# A new non-enzymatic method for isolating human intervertebral disc cells preserves the phenotype of nucleus pulposus cells

**DOI:** 10.1007/s10616-013-9650-7

**Published:** 2013-10-08

**Authors:** Xinyan Tang, William J. Richardson, Robert D. Fitch, Christopher R. Brown, Robert E. Isaacs, Jun Chen

**Affiliations:** 1 Department of Biomedical Engineering, Duke University, Durham, NC USA; 2 Department of Orthopedic Surgery, Duke University Medical Center, Box 3093, 375 Medical Science Research Building, Durham, NC 27710 USA; 3 Department of Surgery, Duke University Medical Center, Durham, NC USA

**Keywords:** Nucleus pulposus, Tissue culture, Phenotype, Intervertebral disc, Integrin

## Abstract

Cells isolated from intervertebral disc (IVD) tissues of human surgical samples are one of potential sources for the IVD cellular therapy. The purpose of this study was to develop a new non-enzymatic method, “tissue incubation”, for isolating human IVD cells. The IVD tissues of annulus fibrosus (AF) and nucleus pulposus (NP) were incubated separately in tissue culture flasks with culture medium. After 7–10 days incubation, cells were able to migrate out of IVD tissues and proliferate in vitro. After 3–4 weeks culture, expanded cells were harvested by trypsinization, and the remaining tissues were transferred to a new flask for another round of incubation. The molecular phenotype of IVD cells from juvenile and adult human samples was evaluated by both flow cytometry analysis and immunocytochemical staining for the expression of protein markers of NP cells (CD24, CD54, CD239, integrin *α*6 and laminin *α*5). Flow cytometry confirmed that both AF and NP cells of all ages positively expressed CD54 and integrin *α*6, with higher expression levels in NP cells than in AF cells for the juvenile group sample. However, CD24 expression was only found in juvenile NP cells, and not in AF or older disc cells. Similar expression patterns for NP markers were also confirmed by immunocytochemistry. In summary, this new non-enzymatic tissue incubation method for cell isolation preserves molecular phenotypic markers of NP cells and may provide a valuable cell source for the study of NP regeneration strategies.

## Introduction

The intervertebral disc (IVD) is the largest avascular structure in the body and is composed of three morphologically distinct regions, the central nucleus pulposus (NP), the peripheral annulus fibrosus (AF), and the cartilaginous endplates. Disc degeneration is considered to be one of the major causes of low back pain, and characterized by dysfunctional cells along with the loss of morphological distinction between regions and extracellular matrix production (Freemont 2009; Urban and Roberts 2003), which ultimately disrupts the finely balanced biomechanics of the disc and spine as a whole (Urban and McMullin 1985; Butler et al. 1990).

Due to a decline of cellularity and potentially a change in cell phenotype with age, the IVD exhibits a very limited capability for self-repair (Smith and Walmsley 1951; Melrose et al. 1992; Zhao et al. 2007). Recently, cell therapy has been used as a strategy to regenerate disc structure and restore disc function (Sakai et al. 2005; Smith et al. 2011; Risbud et al. 2004), and there is significant interest in developing strategies to repopulate the degenerated disc using an appropriate cell source, with work investigating bone marrow-derived mesenchymal stem cells (MSCs), allogeneic chondrocytes and autologous disc cells as potential candidates for NP cell therapies (Sakai et al. 2003; Ganey et al. 2003; Huang et al. 2013; Acosta et al. 2011; McCanless et al. 2011; Yoshikawa et al. 2010; Gruber et al. 2002). A potential cell source for autologous transplantation also includes human disc cells from tissue surgical samples from discectomy procedure. The standard approach for isolating cells from IVD tissue involves enzymatic digestion via pronase and collagenase treatment (Wang et al. 2001; Chen et al. 2002; 2004; Gilchrist et al. 2007; Gabr et al. 2011). However, it is a challenge to isolate disc cells via traditional enzymatic methods because the cells populating human IVD tissues are sparsely distributed and embedded within a very dense extracellular matrix (i.e. collagen and proteoglycan) network. In order to obtain sufficient cell numbers, large amounts of tissue are typically needed (usually pooled from multiple disc levels). Additionally, cells isolated via enzymatic digestion may suffer damage to cell surface receptors immediately upon isolation (Gilchrist et al. 2007), require longer expansion time to recover, and need multiple passages to achieve sufficient cell numbers, with increased passages and longer expansion resulting in cell dedifferentiation (Wang et al. 2001). To overcome these disadvantages, we have developed a new non-enzymatic method, “tissue incubation”, for isolating disc cells. In this study, we use this method to isolate cells from IVD tissues and examine whether NP cell phenotype is preserved, utilizing NP phenotypic markers (CD24, CD54, CD239, laminin α5 and integrin α6) previously validated in our lab (Gilchrist et al. 2007; Gabr et al. 2011; Chen et al. 2006; 2009; Tang et al. 2012). Our findings suggest that this method is effective for isolating phenotypically distinct NP cells for in vitro investigations of disc cell biology and their application in cell-based regenerative medicine.

## Materials and methods

### IVD tissue isolation and incubation

**Fig. 1:**
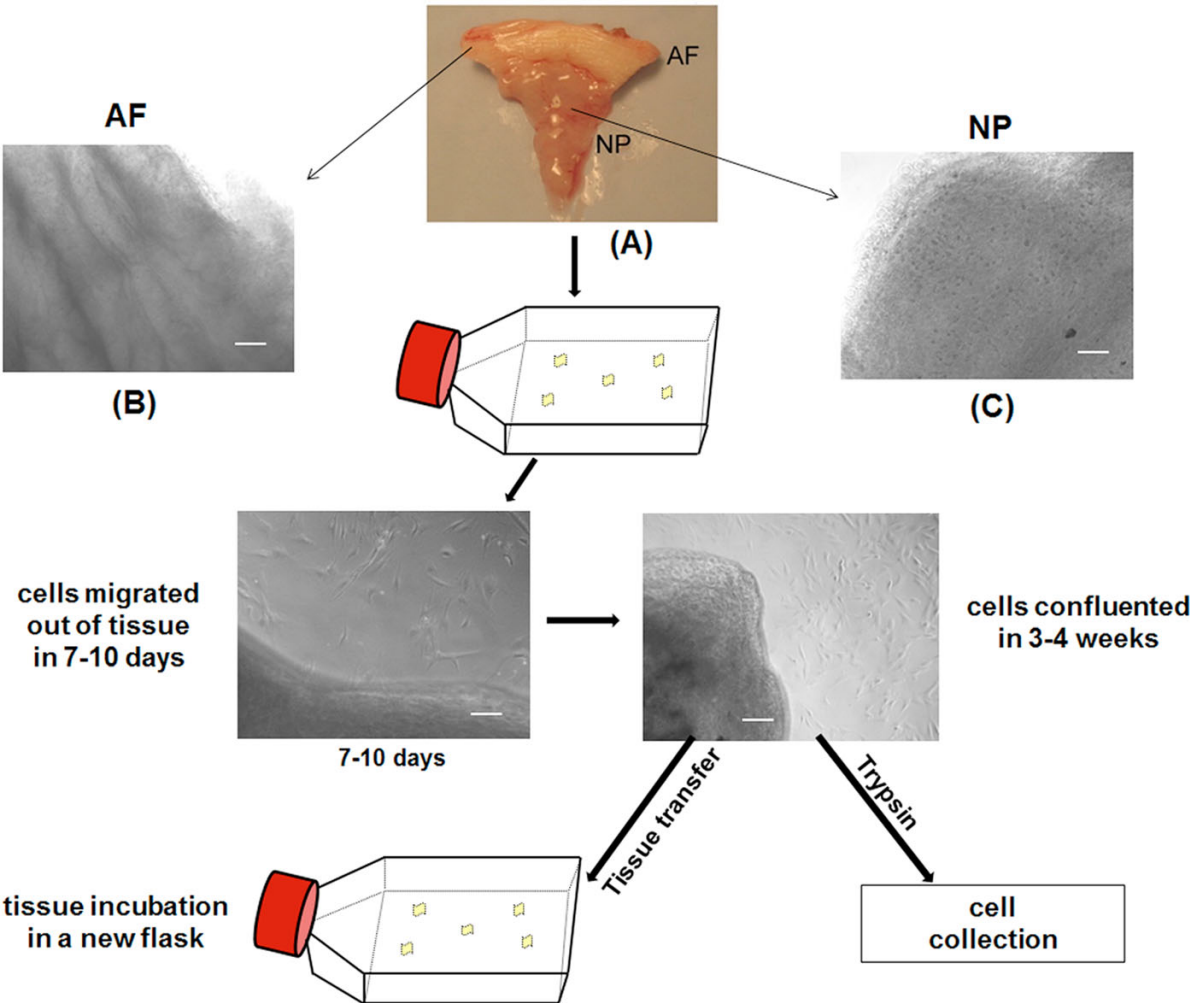
Schematic of human IVD cell isolation via the tissue incubation method. **A** Morphology of AF and NP tissues from IVDs of juvenile patients. Microscopic images of **B** AF tissue and **C** NP tissue used in the tissue incubation. *Bar* 100 μm

Human lumbar IVD tissues (to-be-discarded surgical waste, approved from review by the Duke University Institutional Review Board) were obtained from patients undergoing surgery for degenerative disc disease (total *n* = 4 patients, age 39–71 years old) or scoliosis (total *n* = 4 patients, age 6–21 years old). Tissues were anonymized, with only data for patient age, gender and race were recorded. Disc tissues were rinsed with PBS (EMD Chemicals, Gibbstown, NJ, USA) and grossly separated into AF and nucleus NP according to the anatomic appearance (Gabr et al. 2011). Any other non-disc materials such as endplate bone or cartilage in the surgical sample were discarded prior to tissue incubation. Separated AF and NP tissues were further washed with washing medium (DMEM basal medium with 100 μg/ml kanamycin (Sigma, St. Louis, MO, USA) and 165 μg/ml gentamycin (Gibco, Grand Island, NY, USA), 1.25 μg/ml fungizone (Gibco) three times and cut into small pieces (average size of the tissue explant is 1–2 mm^3^ for AF, 3–5 mm^3^ for NP), then placed in culture medium (F12, Invitrogen Life Technologies, Carlsbad, CA, USA) supplemented with 10 % FBS (Hyclone, South Logan, Utah, USA) in 25-cm^2^ flask coated with 0.1 % gelatin (Sigma) at 37 °C, 5 % CO_2_ condition. Culture medium was changed every 2 days. Once cells had migrated out of tissue and expanded for 3–4 weeks (to about 80 % of confluence), tissue explants were transferred into a new flask for another round of incubation, with remaining cells in the original flask ready for harvesting (see the outline of this method in Fig. 1). In general, incubated tissues can be transferred up to 10 times or until no more cells migrated out.

### Cell harvesting and flow cytometry

Following tissue incubation, disc cells were detached from the culture surface using 0.025 % Trypsin/EDTA (Lonza, Basel, Switzerland) for a very short period of time (<3 min). Total cell number in each flask was counted by the trypan blue assay. The trypsinized cells were allowed to recover in culture medium (F12 medium with 10 % FBS) for 30 min at 37 °C before flow cytometry analysis in order to minimize the possible damage of cell surface receptors due to trypsin. Cells (0.2–0.5 × 10^6^) were then incubated with anti-human antibodies for NP markers (CD24, CD54 and integrin α6, see Table 1) and appropriate isotype controls (mouse or rat IgG, Table 1) for 30 min. Cells were washed twice in PBS and then incubated with appropriate AlexaFluor 488-conjugated secondary antibodies (Invitrogen, Eugene, OR, USA) for 30 min. The percentage of positive cells (%) and mean fluorescence intensity (MFI) for each marker protein were analyzed by flow cytometry (Accuri C6, BD Accuri Cytometers Inc., Ann Arbor, MI, USA).

**Table 1:** Antibodies of markers for human NP cells used in flow cytometry analysis (FC ) and immunocytochemical staining (IC)

Anti-human antibody	Order number (vendor)	Host monoclonal	Isotype control (vendor)	Application
CD24	555,426 (BD Biosciences, San Jose, CA, USA)	mouse	Ms IgG2a, k (BD Biosciences)	IC and FC
CD54	MCA1615GA (AbD Serotec, Raleigh, NC, USA)	mouse	Ms IgG1 (Millipore)	IC and FC
CD239	3,706-1 (Epitomics, Burlingam, CA, USA)	rabbit	Rb IgG (Epitomics)	IC
Laminin α5	MAB1924 (Millipore, Billerica, MA, USA)	mouse	Ms IgG2a (AbD Serotec)	IC
Integrin α6 (CD49f)	555,734 (GoH3) (BD Biosciences)	rat	rat IgG2a (BD Biosciences)	IC and FC

### Immunocytochemical staining

In order to assess expression of NP markers in human disc cells under monolayer culture conditions, NP and AF cells were trypsinized and seeded onto 8-well chamber slides (Nalge Nunc, Rochester, NY, USA, 20,000 cells/well) coated with 0.1 % gelatin. Cells were incubated in culture medium overnight at 37 °C to allow for attachment, followed by fixation in 4 % formaldehyde (Electron Microscopy Sciences, Hatfield, PA, USA) and incubation with a blocking solution (30 min), washing with PBS, and incubation with anti-human antibodies for specific NP markers (CD24, CD54, C239, laminin α5 and integrin α6, see Table 1) for 2 h. For control sections, appropriate mouse, rat or rabbit IgG isotype controls (Table 1) were used. All sections were then incubated with appropriate AlexaFluor 488-conjugated secondary antibodies (Invitrogen) for 30 min in blocking solution, counter-stained with propidium iodide (0.2 mg/ml, Sigma) to label cell nuclei, and imaged via confocal laser scanning microscopy (Zeiss LSM 510; 20× NA 0.5 and 63× NA 1.2 objectives; Carl Zeiss, Thornwood, NY, USA).

## Results

### IVD cells release from tissue explants

**Fig. 2:**
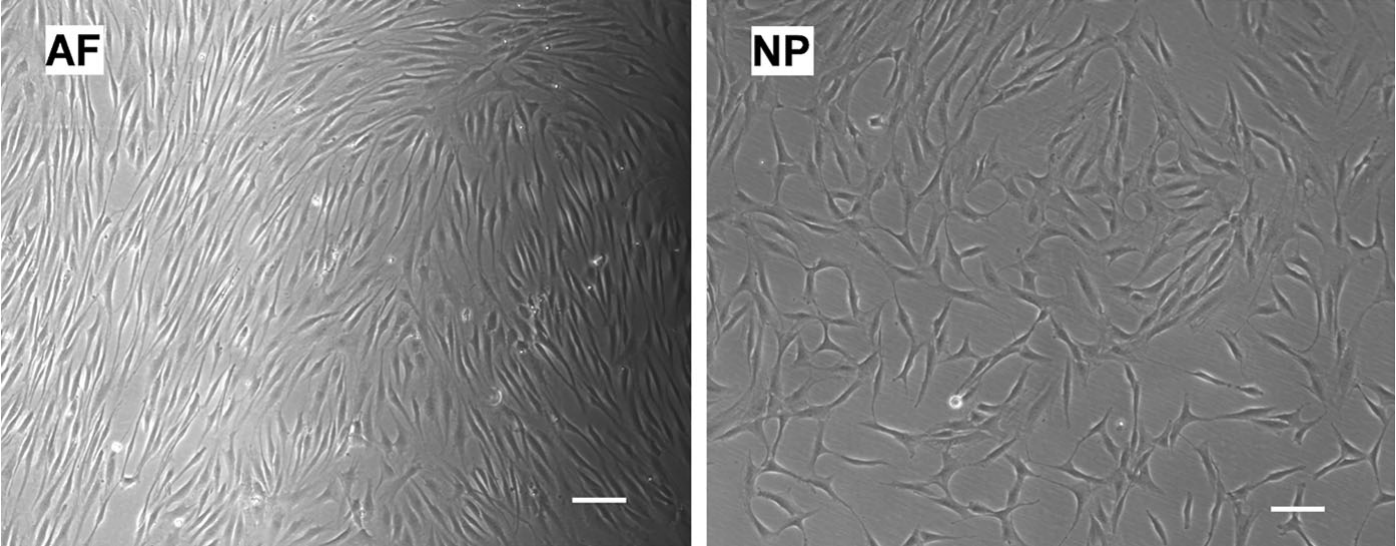
Morphology of cells migrated out of IVD tissues. *Left*: AF cells from tissue incubation (*elongated shape*). *Right*: NP cells from tissue incubation (*spindle shape*). *Bar* 100 μm

AF and NP tissues harvested from surgical samples generally displayed different tissue morphology and structure. A distinct oriented collagen fiber-like structure was observed in AF tissues (Fig. 1A, B), while NP tissues of juvenile discs exhibited a gelatinous-like structure and did not have oriented collagen fiber structure (Fig. 1A, C). After 7–10 days of incubation, cells started to migrate out of tissues (Fig. 1). It was observed that AF cells generally migrated out of tissue earlier than NP cells, and that tissue from young patients also started “releasing” cells earlier as compared to that of aged tissue. In all ages, released NP cells displayed spindle morphology, whereas released AF cells exhibited a more elongated shape on the culture surface (Fig. 2). Generally, after 3–4 weeks of incubation, approximately 0.5 × 10^6^ cells per flask were collected from juvenile AF and NP disc tissue. For the adult disc tissue, however, lower numbers of cells (AF ~0.3 × 10^6^ cells/flask, NP ~0.2 × 10^6^ cells/flask) were collected (Table 2). This finding of lower cell yield in aged tissue is consistent with a previous report showing the lower cellularity in aged IVD (Zhao et al. 2007).

**Table 2:** Average total number of cells migrated out of human IVD tissues after one round of tissue incubation

Age (*n* = 4 each group)	Quantity of tissues/flask	Cell number (×10^6^ ± SD)	
		AF	NP
Juvenile	5 pieces	0.5 ± 0.38	0.5 ± 0.21
Adult	5 pieces	0.3 ± 0.10	0.2 ± 0.09

### NP phenotype detection by flow cytometry

**Fig. 3:**
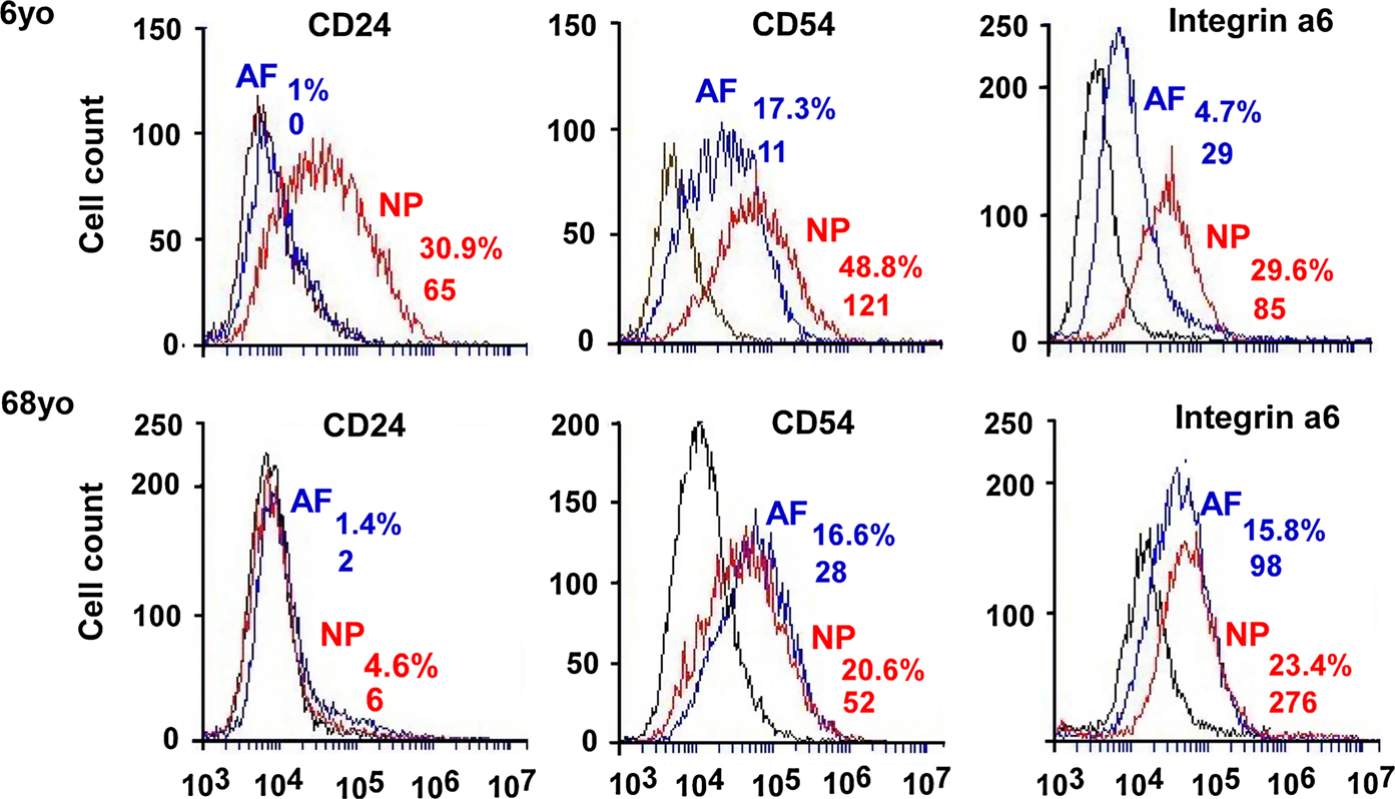
Flow cytometric analysis for NP marker expression in IVD cells from different ages of patients (6 and 68 year old). Representative histograms of flow cytometry illustrate the relative fluorescence intensity of protein expression on X-axis for migrated cells (cell surface receptors: CD24, CD54 and integrin α6). *Black line*: isotype control, *blue line*: AF cells, *red line*: NP cells. The numbers appearing in each histogram are positive-cell percentage and MFI. (Color figure online)

To confirm the molecular phenotype of NP cells that migrated out of tissues, we evaluated the expression of cell surface receptors (CD24, CD54 and integrin α6) previously reported in NP cells of rat (Tang et al. 2012), pig (Gilchrist et al. 2007) and human (Gabr et al. 2011; Chen et al. 2009). Flow cytometry analysis for cells from IVD samples of young (6-year old) and aged (68-year old) patients showed CD24 was only expressed in 6-year old NP cells while CD54 and integrin α6 expression was found both in AF and NP cells of both 6- and 68-year old samples (Fig. 3). NP cells from the 6-year old sample expressed CD24 with higher percentage of positive cells (30.9 %) and MFI (65) as compared to that of 68-year old sample (4.6 %, MFI 6) (Fig. 3 left). In contrast, CD24 expression was not detected in AF cells of any age (Fig. 3 left). For CD54 expression, NP cells from the 6-year old sample had a higher percentage of positive cells (48.8 %) and MFI (121) as compared to AF cells of 6-year old (17.3 %, MFI 11) (Fig. 3 middle). However, CD54 expression levels in 68-year old AF and NP cells was found to be similar (AF: 16.6 %, MFI 28; NP: 20.6 %, MFI 52) (Fig. 3 middle). For integrin α6, NP cells generally had a higher level expression than AF cells in both young [6-year old: NP (29.6 %, MFI 85); AF (4.7 %, MFI 29)] and old patients [68-year old: NP (23.4 %, 276); AF (15.8 %, MFI: 98)] (Fig. 3 right). In general, we found that both positive cell percentage and MFI displayed the similar expression pattern as mentioned above in all other surgical samples.

### NP phenotype detection by immunocytochemistry

**Fig. 4:**
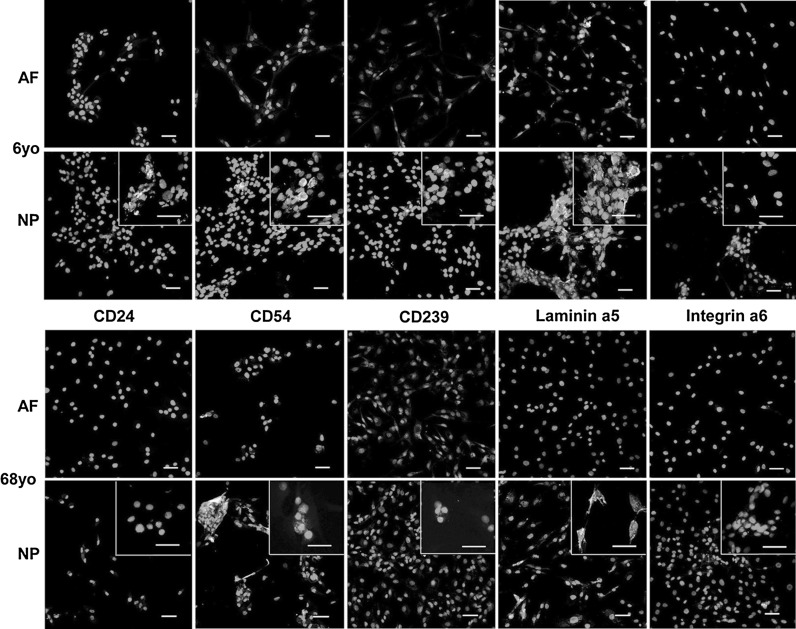
Immunocytochemical staining for NP marker expression in IVD cells (NP, AF) derived from patients (6 and 68 yo). *Bar* 50 μm; yo: year old. Images with higher magnification are presented as the *insets* in lower panels for NP cells

Immunolabeling techniques also were used to confirm protein expression patterns for disc cells during monolayer culture. Similar to flow cytometry findings, immunocytochemical staining confirmed a distinct staining pattern of NP markers in NP cells as compared to AF cells for all samples in both juvenile and old groups. As demonstrated in Fig. 4, NP cells from the 6-year old sample stained intensively positive for cell surface receptors (CD24, CD54, CD239 and integrin α6) and NP-specific extracellular matrix, laminin α5 (Fig. 4). However, NP cells from the 68-year old sample did not express CD24 and stained only slightly positive for CD239 and integrin α6, although expression of CD54 and laminin α5 were still clearly observed (Fig. 4). In contrast, AF cells of both ages were negative for NP markers, with the exception of slightly positive staining for CD54 and laminin α5 in 6-year old AF cells (Fig. 4).

## Discussion

Human IVD tissue is a complicated structure in which the cells are sparsely distributed within a dense extracellular matrix. It is a significant challenge to isolate primary IVD cells, as isolation by traditional enzymatic digestion often results in damage to cell surface receptors, requiring longer times in culture for cells to recover and multiple cell passages to achieve sufficient cell numbers. These longer expansion times and increased passage numbers may result in cell dedifferentiation. In the current study, we successfully isolated human IVD cells with distinct NP cell phenotypes through a non-enzymatic method, termed “tissue incubation”. This method permits successful cell isolations from small amounts of IVD waste tissue, including that obtained from minimally invasive surgery cases where large amounts of tissue are not available. The method also permits us to isolate cells from individual IVD levels, making level-specific studies possible. Furthermore, NP or AF cells isolated by this tissue incubation method may be relatively pure because any monocytes or other immune cells residuals (that possibly have infiltrated degenerated or pathological tissue) could be washed away by the multiple medium change processes during the tissue incubation period. Interestingly, disc cells can be collected continuously through transferring the same tissue to a new culture surface many times. According to our experience, IVD cells could be passaged up to 5–6 times before they stop doublings (we only used cells without passage to prevent cell dedifferentiate in this study). Although IVD cells are belonging to differentiated cells (as a more specialized cell type), they still can be promoted to divide under serum culture condition (10 % FBS) as other primary cell culture in vitro. Importantly, recent studies have revealed that human IVD cells may contain cells exhibiting notochordal cell phonotypes (Chen et al. 2009; Weiler et al. 2010) and progenitor cell population (Risbud et al. 2007; Sakai et al. 2012). This may also explain that human IVD cells may not only accumulate in G1 phase and probably preserve some signature of progenitor cells which could make them grow and multiply in vitro.

We counted the cell numbers migrating out of disc tissues and compared cell “release” differences between tissue regions (AF vs. NP) and ages (juvenile vs. adult). NP cells were much slower to migrate out as compared to AF cells. It is possible that this could be related to differences in tissue attachment to the culture surface, with soft NP tissue not attaching as readily or completely to the flask surface as compared to relatively hard AF tissue. Additionally, juvenile tissues seemed to “release” more cells than adult tissues, which may reflect the higher cellularity in juvenile as compare to the aged tissues. Importantly, our findings indicate that cells released from IVD tissues exhibit distinct cellular morphologies and molecular phenotypes (CD24, CD54, integrin α6, CD239 and laminin α5) for NP cells. This new non-enzymatic method for IVD cell isolation can be used to acquire disc cells with a preserved phenotype, which will be a useful tool for studying IVD cell biology in vitro and exploring possibilities for IVD cellular therapies.

CD24, a glycosylphosphatidylinostitol-anchored cell surface protein that functions in differentiation and activation of granulocytes and B lymphocytes (Nielsen et al. 1997), has been reported to be expressed by NP cells of rat and human chordoma (a notochordal-derived tumor) (Fujita et al. 2005). In our previous study, we also confirmed the NP-specific expression for CD24 in rat IVDs (Tang et al. 2012). Here we further discovered that CD24 was strongly expressed in juvenile human NP cells (6-year old) through flow cytometry and immunocytochemical staining, while no expression was detected in AF and adult NP (68-year old) cells. Importantly, this finding of a CD24 differential expression pattern with age difference suggests that CD24 is an important maker for young human NP cells. CD54 (ICAM-1) is a cell surface glycoprotein expressed in a variety of cell types including endothelial cells, activated leukocytes, infiltrated macrophages (Vachula and Van Epps 1992; Bevilacqua 1993). Previously, we found that human IVD cells also expressed CD54 and its expression could be up-regulated by proinflammatory cytokines (IFN-γ and TNF-α) (Gabr et al. 2011). In this study, CD54 was expressed in both cultured human AF and NP cells of all ages in vitro.

CD239, a receptor that binds exclusively to the laminin α5 chain, and has been previously identified in human red blood cells and as co-receptors in epithelia, endothelia, smooth muscle cells and immature human NP cells (Chen et al. 2009; Kikkawa and Miner 2005). Positive immunocytochemical staining of CD239 was observed in only young NP cells, which is consistent with our previous study that demonstrated the expression pattern of CD239 and laminin α5 in immature disc tissues (Chen et al. 2009). The finding of expression for both laminin α5 chain and CD239 in the immature NP, but not AF, regions points towards the unique and distinctly different developmental origins of the NP and AF (Rufai et al. 1995). These expression patterns could be linked to notochordal origin of the NP tissue based on the known involvement of laminins in the basement membrane surrounding notochord during differentiation (Parsons et al. 2002). Our previous study also found that the expression of α6 integrin subunit, another laminin receptor, is associated with cells of the immature porcine and human NP tissue (Chen et al. 2006; 2009; Nettles et al. 2004), and that NP cell adhesion to laminin-111 substrates is mediated by the α6 integrin (Gilchrist et al. 2007). In this study, we further confirmed distinct expression for the α6 subunit in human NP cells by both flow cytometry analysis and immunocytochemical staining. A similar region-specific expression pattern for laminin α5 (present for NP cells, almost entirely absent in AF cells) was also observed.

In summary, this new non-enzymatic tissue incubation method for human IVD cell isolation is simple, efficient, and preserves the molecular phenotype (CD24, CD54, CD239, integrin α6 and laminin α5) of NP cells. Cells isolated via this method may provide a pure cell source for the study of disc degeneration mechanisms, NP cell biology, and tissue engineering and regeneration strategies.
